# Tumor cell invasion of von Hippel Lindau renal cell carcinoma cells is mediated by membrane type-1 matrix metalloproteinase

**DOI:** 10.1186/1476-4598-5-66

**Published:** 2006-12-01

**Authors:** Brenda L Petrella, Constance E Brinckerhoff

**Affiliations:** 1 Department of Medicine, Norris Cotton Cancer Center, Dartmouth Medical School, Lebanon, NH, USA

## Abstract

**Background:**

Metastatic renal cell carcinoma (RCC) remains the leading cause of mortality in patients with clear cell RCC arising from mutations in the von Hippel Lindau (VHL) tumor suppressor. Successful RCC tumor suppression by VHL requires the negative regulation of hypoxia inducible factor alpha (HIF alpha) protein and its downstream targets. Thus, identification of HIF target genes responsible for RCC tumor progression will aid in the development of therapies for this disease. We previously identified membrane type-1 matrix metalloproteinase (MT1-MMP) as a transcriptional target of HIF-2alpha in RCC cells null for VHL and showed that MT1-MMP is overexpressed in these cells. MT1-MMP is a key regulator of tumor progression through its functions as a matrix-degrading enzyme, as well as its ability to cleave factors, such as adhesion molecules and other MMPs. The aim of this study was to investigate the contribution of MT1-MMP to the invasive potential of RCC cells using *in vitro *type I collagen degradation and invasion assays.

**Results:**

We evaluated RCC cells wild-type (WT8) and null (pRc-9) for VHL for invasive characteristics and showed that the pRc-9 cells demonstrated a greater propensity for both invasion and degradation of a type I collagen matrix. Furthermore, overexpression of either HIF-2alpha or MT1-MMP in the poorly invasive cell line, WT8, promoted collagen degradation and invasion of these cells. Finally, using RNAi, we show that inhibition of MT1-MMP suppresses tumor cell invasion of RCC cells.

**Conclusion:**

Our results suggest that MT1-MMP is a major mediator of tumor cell invasiveness and type I collagen degradation by VHL RCC cells that express either MT1-MMP or HIF-2alpha. As such, MT1-MMP may represent a novel target for anti-invasion therapy for this disease.

## Background

Kidney cancer represents ~3% of cancer deaths worldwide and is the most deadly of the common urological diseases [1]. While combined nephrectomy and immunotherapy are standard care for localized primary renal cell carcinoma (RCC) tumors, approximately 30% of these treated patients will eventually develop metastases [2]. Additionally, one third of patients present with metastatic disease at time of diagnosis of the RCC primary tumor [3]. Treatment of metastatic RCC remains difficult primarily due to the resistance of the tumors to adjuvant and immunotherapies [4]. With the median survival of metastatic RCC patients being less than one year, investigation into more effective anti-metastatic therapies is clearly warranted [3,5-7].

Renal cell carcinoma is classified into four histological subtypes, including clear cell, papillary, chromophobe, and collecting duct [2]. Almost 80% of sporadic RCC is of the clear cell subtype and results from inactivation of the tumor suppressor, von Hippel Lindau (VHL) [8]. Loss of VHL function also manifests itself as a dominantly inherited familial cancer syndrome, impacting several organ systems [8,9]. Life expectancy is greatly reduced for ~40% of VHL patients who develop RCC, most commonly due to complications from metastatic disease [5,6,10,11].

The most well-characterized function of VHL is in controlling the oxygen-sensing mechanism of the cell through its regulation of hypoxia inducible factor (HIF) alpha subunits (1α,-2α,-3α) [10,12]. HIF is a heterodimeric transcription factor consisting of two subunits, HIF-α and HIF-β [13]. While the β-subunit of HIF is constitutively expressed, the HIF-α protein is labile and detectable only under hypoxic conditions or when VHL is inactivated [8,13-15]. Under normoxic conditions, VHL negatively regulates the levels HIF-α subunit through ubiquitin-targeted protein degradation [16,17]. Thus, inactivation of VHL in RCC is associated with increased levels of HIF-α isoforms and a subsequent increase in hypoxia-inducible genes, such as those involved in angiogenesis (VEGF, PDGF), erythropoiesis (EPO), glycolysis (Glut1), cell growth and survival (Cyclin G2, TGF-α), and cell migration (CXCR4), suggesting that the genes upregulated by the VHL-HIF pathway are involved in the progression of renal cell carcinoma [8,13,15].

Although the majority of VHL mutations abrogate the regulation of HIF-α protein, a few mutations exist that retain the ability for VHL to regulate HIF-α, and these mutations are not associated with the formation of RCC [9,18]. In fact, expression of such a VHL mutant, which retains the ability to negatively regulate HIF-2α, suppresses tumor formation of VHL null RCC cells *in vivo *[18]. These results demonstrate that successful tumor suppression in renal cells depends on the proper regulation of HIF-2α rather than on the presence of VHL. Further evidence suggests that HIF-2α, rather than HIF-1α, is the VHL target responsible for tumorigenesis [19]. Indeed, inhibition of HIF-2α is required for tumor suppression by VHL in RCC *in vivo *[20-22]. These findings illustrate the importance of understanding the various roles that HIF-2α targets play in renal cell tumorigenesis.

Previously, we identified membrane-type 1 matrix metalloproteinase (MT1-MMP) as a target gene of HIF-2α in RCC cells mutant for VHL [23]. MT1-MMP is a membrane bound member of the family of zinc-dependent endopeptidases known as the matrix metalloproteinases (MMPs), which function in remodeling the extracellular matrix (ECM) [24,25]. Due to their vast repertoire of substrates and functions in normal cellular processes, MMPs are strictly regulated to guarantee appropriate, homeostatic proteolytic events [26,27]. Elevated levels of MMPs have been linked to the invasive behavior of most human cancers as well as to other characteristics of tumors [25,26,28]. In addition to pericellular proteolysis of type I collagen, MT1-MMP is known to control cell-ECM contacts, localize to the leading edge of invasive cells, cleave adhesion molecules, and activate latent MMP-2 and MMP-13 [24]. As a result, MT1-MMP plays multiple roles in tumorigenesis, including tumor invasion, regulation of tumor cell growth, cell migration, and angiogenesis [29,30]. Our previous data suggest that the loss of the VHL tumor suppressor, and subsequent stability of HIF-2α, leads to the induction of MT1-MMP expression in RCC. Interestingly, MT1-MMP expression has been linked to advanced stages of RCC [31,32].

Given the important role of MT1-MMP in the progression of other cancers [24], we hypothesized that as a HIF-2α target, MT1-MMP may play a role in the progression of VHL-/- RCC tumor invasion to metastatic disease. A metastatic tumor cell must invade through two main extracellular matrix barriers: interstitial collagen in the stromal environment (comprised mainly of type I collagen) and basement membrane (comprised primarily of type IV collagen) [33]. Koochekpour *et al*. showed *in vitro *that VHL-/- RCC cell invasion of type IV collagen is enhanced by the addition of neutralizing antibodies to TIMPs (tissue inhibitors of matrix metalloproteinases), the natural inhibitors of MMP activity, thereby implicating a role of MMPs in this invasion [34]. These authors also showed that VHL mutant RCC cells overexpress the gelatinases, MMP-2 and MMP-9, which function to degrade type IV collagen found in basement membrane. In our previous studies, we reported that MT1-MMP is the main type I collagenolytic enzyme expressed by VHL null RCC cells, suggesting that this enzyme may mediate RCC invasion of type I collagen [23]. Therefore, in this report, we specifically investigated the role of MT1-MMP in VHL RCC tumor cell invasion using *in vitro *assays to measure the ability of the cells to degrade type I collagen and to invade through a type I collagen matrix. Using gene overexpression studies and RNAi, our data directly link HIF-2α and MT1-MMP expression to an invasive phenotype of RCC cells, and targeted inhibition of MT1-MMP is required to block this invasion. We conclude that MT1-MMP is the primary mediator of both tumor cell invasion and degradation of type I collagen in these RCC cells and may represent an effective target for the treatment of invasive renal cell carcinoma.

## Results

### VHL expression inhibits invasive properties of RCC cells

In previous studies [23], we used human RCC cells derived from a VHL null parental cell line (786-0), which was stably transfected with the pRc/CMV vector expressing a wild-type copy of VHL (WT8) or with the vector alone (pRc-9) [16]. Notably, these cells only express HIF-2α and do not express the HIF-1α isoform [17]. We determined that the lack of VHL tumor suppressor activity stabilized HIF-2α protein and increased MT1-MMP expression through direct transcriptional transactivation by HIF-2α (summarized in Table 1) [23]. These data provide a link between the loss of VHL function and the overexpression of an enzyme responsible for pericellular proteolysis of type I collagen and the activation of pro-MMP-2 [23,35,36]. In addition, our studies showed that MT1-MMP is the primary type I collagenolytic enzyme expressed by the VHL null cells. MMP-2 and MMP-9, which degrade type IV collagen in basement membrane, are also upregulated in these cells [23,34]. Since metastatic tumor cells must invade through both interstitial collagen in the stromal environment and basement membrane [33], we hypothesize that MT1-MMP may contribute to the invasiveness of renal cell carcinoma in the context of VHL inactivation by mediating type I collagen invasion.

**Table 1:** Renal cell carcinoma cell lines

**Designation**	**Transfectant**	**VHL status**	**HIF-2α protein**	MT1-MMP levels*
786-0	none	null	stable	+++
WT8	pRc/CMV-VHL	wild-type	not detectable	+
pRc-9	pRc/CMV-empty	null	stable	+++

* Petrella, et al. 2005

"+" and "+++" indicate relative mRNA expression levels between cell lines

**Figure 1:**
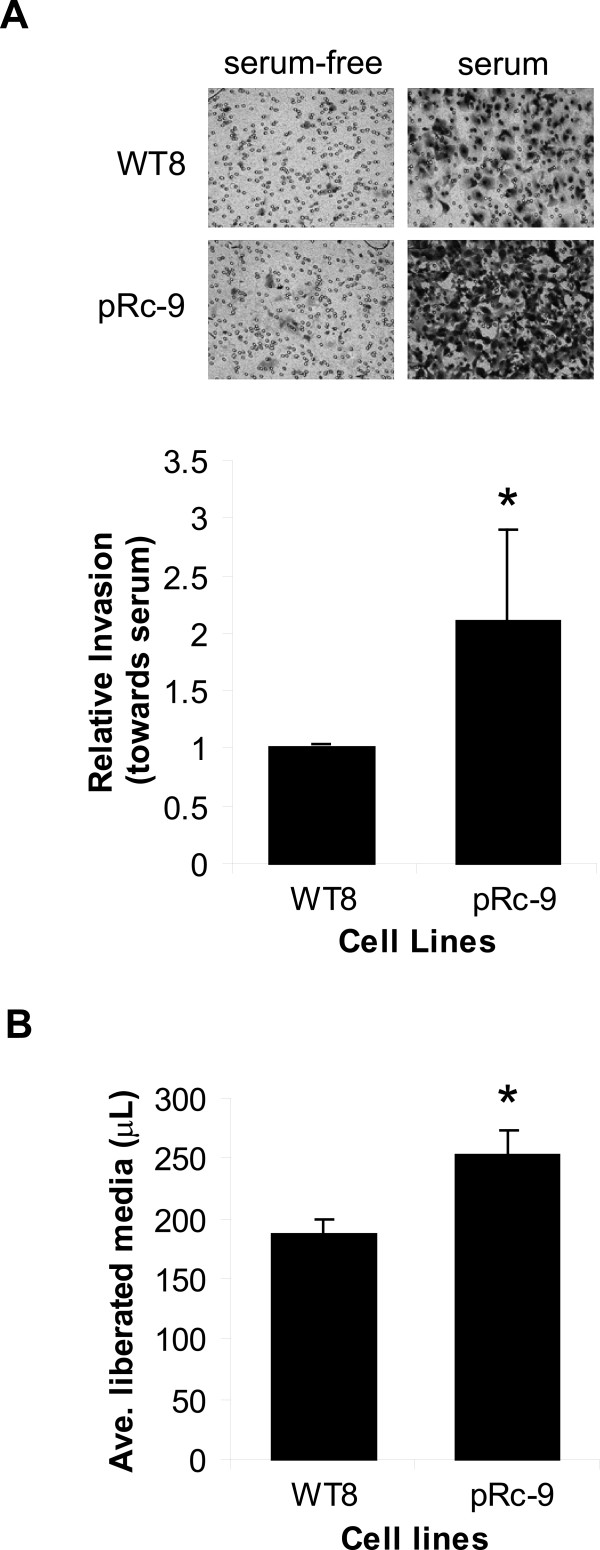
**Expression of VHL inhibits collagen invasion and degradation**. **A**. Collagen invasion. WT8 and pRc-9 cells were cultured in serum-free media on top of type I collagen coated membranes in a Transwell^® ^invasion assay system. The lower chambers contained either serum-free DMEM as a negative control or DMEM supplemented with 10% FBS as a chemoattractant. At time of harvest, the non-invaded cells and collagen matrix on top of the membranes were removed, and invaded cells on the bottoms of the membranes were stained with methylene blue. A representative picture is shown of cells invading towards serum-free versus serum-containing media. Cells were counted from 3 fields per sample at 100× and averaged. Values in the graph represent the average number of cells invaded towards serum from four separate experiments relative to WT8 cell invasion (mean+/-S.D.); *P *< 0.05 (*). **B**. Collagen degradation. WT8 and pRc-9 cells were serum-starved overnight, harvested and then embedded in a mixture of type I collagen and serum-free DMEM for the collagen degradation assay. The collagen gel was allowed to solidify and serum-free media was added to the top of the collagen gel. The overlying media was weighed 48 hours after time of plating, and collagen degradation was determined by the volume of media liberated from the collagen gel. Values represent the average μL of media released from three samples (mean+/-S.D.); *P *< 0.01 (*) and are representative of four separate experiments. Statistical analyses were performed using the student's *t*-test.

To begin our investigations, we characterized the WT8 and pRc-9 cells for differences in tumor cell invasion and matrix degradation that would suggest a role for MT1-MMP [24,30,35]. We first compared the ability of the WT8 and pRc-9 cells to invade through a type I collagen matrix using a Transwell^® ^insert assay system. Neither cell line invaded towards serum-free media. However, the pRc-9 cells, mutant for VHL, invaded approximately twice as effectively towards a serum chemoattractant when compared to the WT8 cells (Figure 1A). These data suggest that VHL expression suppresses tumor cell invasion through type I collagen. In similar *in vitro *invasion assays using Matrigel or type IV collagen, RCC cells mutant for VHL were also shown to be more invasive than RCC cells expressing wild-type VHL [34,37,38].

Invasive tumor cells have the ability to migrate and to remodel the surrounding, restrictive ECM using enzymes, such as MT1-MMP, for pericellular proteolysis [36,39]. To ask whether the observed increase in cell invasion by the pRc-9 cells involves increased matrix degradation, we measured the ability of these cells to degrade a type I collagen matrix using an *in vitro *collagen degradation assay. This assay is based upon the fact that when a fibrillar collagen gel is degraded by cells embedded in it, culture medium is liberated. This medium can be recovered and weighed, and the amount of medium liberated provides a simple, but accurate, measure of matrix destruction [40,41]. Indeed, we found that collagen degradation was increased by approximately 25% (*P *< 0.005) in the VHL null cell line, pRc-9, as compared to the WT8 cells expressing wild-type VHL, indicating that the lack of VHL function promotes type I collagen degradation (Figure 1B). A similar increase in collagen degradation was observed in the VHL null 786-0 parental line (data not shown).

### Expression of HIF-2α or MT1-MMP is sufficient to increase the invasive potential of RCC cells wild-type for VHL

**Figure 2:**
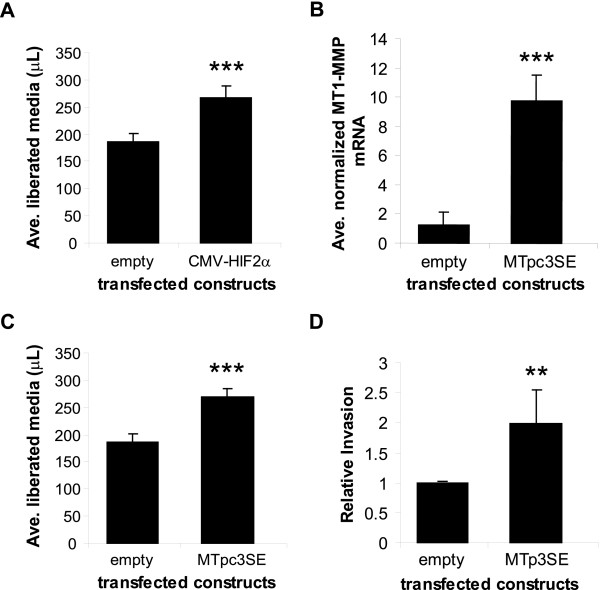
**Expression of HIF-2α or MT1-MMP in WT8 cells increases matrix degradation and invasion**. **A**. Collagen degradation. WT8 cells were transfected with either a control empty vector or pCMV-HIF-2α. Transfectants were serum-starved overnight, harvested and then embedded in a mixture of type I collagen and serum-free DMEM for the collagen degradation assay as in Fig 1. After 48 hours, the overlying media was weighed, and collagen degradation was determined by the volume of media liberated from the collagen gel. Values represent the average μL of media released from six samples (mean+/-S.D.); *P *< 0.0001 (***). **B**. Quantitative real-time RT-PCR analysis of MT1-MMP mRNA expression in WT8 cells co-transfected with pCMV-eGFP and either a control empty vector or MTpc3SE. Values represent the average pg of MT1-MMP mRNA normalized to ng of GFP mRNA from three separate transfections (mean+/-S.D.); *P *< 0.0001 (***). **C**. Collagen degradation. WT8 cells were transfected with either a control empty vector or MTpc3SE. Transfectants were subjected to the collagen degradation assay as in Fig 1. After 48 hours, the overlying media was weighed, and collagen degradation was determined by the volume of media liberated from the collagen gel. Values represent the average μL of media released from six samples (mean+/-S.D.); *P *< 0.0001 (***). **D**. Collagen invasion. WT8 cells were transfected with either an empty vector or MTpc3SE. Transfectants were subjected to a collagen invasion assay as described for Fig 1. At time of harvest, the non-invaded cells and collagen matrix on top of the membranes were removed and membranes stained with methylene blue. Cells were counted from 3 fields per sample at 100× and averaged. Values represent the average number of invaded cells from five separate experiments set as relative cell invasion of the empty vector transfectant (mean+/-S.D.); *P *< 0.005 (**). Statistical analyses were performed using the student's *t*-test.

Although the most well-described function of VHL is the negative regulation of HIF-α isoforms, VHL has many HIF-independent functions as well, including assembly of actin filaments [42], fibronectin matrix assembly [38,43,44], regulation of PKC isotypes [37], and endocytosis of FGFR1 [45]. Each of these VHL functions has been implicated in either RCC cell migration (cell movement in the absence of matrix) or invasion (cell movement through a matrix). To test whether the increased invasion and degradation of type I collagen observed in VHL null cells was due to the overexpression of HIF-2α target genes or to the loss of HIF-independent functions of VHL, we overexpressed HIF-2α in the context of wild-type VHL. Previously, we showed that transient overexpression of pCMV-HIF-2α in the WT8 cells was sufficient to drive transcription of a reporter construct containing HIF binding sites even in the presence of wild-type VHL [23]. Therefore, we introduced a pCMV-HIF-2α expression construct into the WT8 cells by transient transfection and measured the ability of these transfectants to degrade a collagen matrix. As shown in Figure 2A, overexpression of HIF-2α in the WT8 cells increased collagen degradation of these cells by ~30% (*P *< 0.0001), similar to the difference in collagen destruction between the WT8 and the pRc-9 cells (see Figure 1B). These results indicate that HIF-2α expression is sufficient to increase the collagen-degrading ability of cells that are wild-type for VHL, thereby suggesting that the increased invasion and matrix degradation measured in the pRc-9 cells may be due to aberrant expression of a HIF target gene(s) rather than the dysregulation of other VHL targets. These data support our hypothesis that as a target of HIF-2, MT1-MMP may contribute to VHL RCC tumor cell invasion.

To further test this hypothesis, we transiently transfected an MT1-MMP expression construct (MTpc3SE) into the WT8 cells and measured the ability of these transfectants to degrade type I collagen. This experimental design allowed us to study the effects of MT1-MMP expression on collagen invasion in the context of wild-type VHL and HIF-2α instability, thereby eliminating the contribution of other factors related to VHL mutations. Increased expression of MT1-MMP in the MTpc3SE transfected cells was confirmed by real-time RT-PCR, and MT1-MMP mRNA levels were approximately six-fold greater than the empty vector control transfectants (Figure 2B). The difference in MT1-MMP levels in these transfectants is comparable to the difference in endogenous MT1-MMP levels in the WT8 and pRc-9 cells previously published [23]. Furthermore, overexpression of MT1-MMP increased collagen degradation of the WT8 cells by approximately 30% (*P *< 0.0001; Figure 2C), again resembling the increase in degradation measured in the pRc-9 cells as well as the HIF-2α transfected WT8 cells (see Figures 1B, 2A). These data indicate that MT1-MMP may be the HIF target gene responsible for the increased collagen degradation by the VHL mutant cells since, to our knowledge, no other HIF target with the ability to cleave type I collagen has been identified in these cells.

Next, we tested whether MT1-MMP expression was sufficient to promote collagen invasion of the WT8 cells by transient expression of the MTpc3SE construct in these cells and measured the ability of the transfectants to invade through a type I collagen matrix using the assay system as described in Figure 1. Expression of MT1-MMP in the WT8 cells increased collagen invasion approximately two-fold compared to empty vector transfected cells, showing that exogenous MT1-MMP expression alone drives the invasiveness of cells that retain VHL function (*P *< 0.005; Figure 2D). Importantly, the difference in invasiveness between the WT8 transfectants mimics the difference in collagen invasion observed between the WT8 and pRc-9 cells (see Figure 1A). Together with our previous finding that MT1-MMP expression is regulated by HIF-2α in VHL null cells [23], our data suggest that one mechanism by which VHL may inhibit tumor invasion is by suppressing MT1-MMP expression through the negative regulation of HIF-2α protein.

### Inhibition of MT1-MMP is necessary to decrease invasive properties of RCC cells expressing MT1-MMP or HIF-2α

To extend these findings, we asked if MT1-MMP was necessary for VHL RCC cell invasion of collagen using RNAi to specifically inhibit MT1-MMP expression. We could not use the pRc-9 cell line for these experiments due to the sensitivity of these cells to transfection and subsequent manipulations. Although expression of MT1-MMP was effectively reduced by three different targeting siRNAs in the pRc-9 cells (data not shown), once transfected, the cells did not survive trypsinization and culturing on top of collagen for use in the invasion assays.

**Figure 3:**
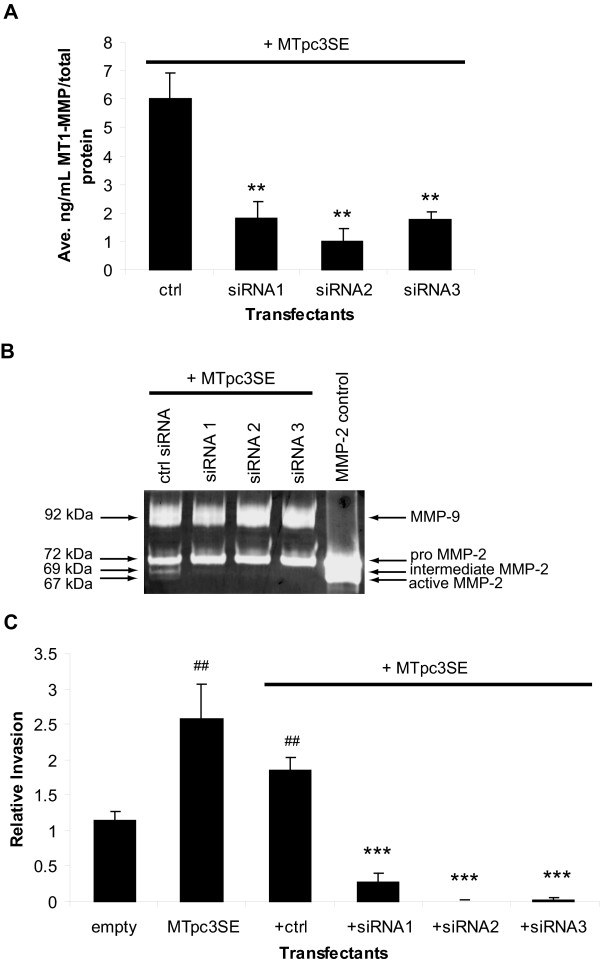
**Inhibition of MT1-MMP blocks RCC tumor cell invasion**. **(A) and (B) **WT8 cells were transfected for 48 hours with MTpc3SE and control or 3 specific MT1-MMP siRNA oligos. Each transfectant was additionally co-transfected with pCMV-eGFP. Transfection efficiency was consistent among the transfectants and was approximately 50%. **A**. MT1-MMP protein expression in the transfectants as quantitated by an MT1-MMP ELISA activity assay. Values represent the average [ng/mL] MT1-MMP of three transfections normalized to [μg/mL] total protein and are representative of three experiments (mean+/-S.D.); *P *< 0.005 (**) compared to control siRNA. **B**. Gelatin zymography of conditioned media from transfectants to measure the presence of active MMP-2. Cells were transfected overnight and then cultured in serum-free media to condition the media for 24 hours. Conditioned media was concentrated before analysis of gelatinolytic activity. **C**. *In vitro *invasion assay using type I collagen coated Fluoroblok™ membrane inserts. WT8 cells were transfected for 48 hours with a control empty vector, MTpc3SE alone, or MTpc3SE and control or 3 specific MT1-MMP siRNA oligos. Each transfectant was additionally co-transfected with pCMV-eGFP and transfection efficiency was approximately 50%. Transfectants were serum-starved overnight before culturing on top of the collagen coated membranes in serum-free media. The lower chambers contained DMEM supplemented with 10% FBS as a chemoattractant. Invaded cells were viewed by GFP fluorescence and counted from the entire membranes at 40×. Values represent the average number of invaded cells from three separate transfections set as relative invasion of the empty vector control and are representative of two experiments (mean+/-S.D.); *P *< 0.005 (##) compared to empty vector control; *P *< 0.0005 (***) compared to control siRNA. Statistical analyses were performed using the student's *t*-test.

Alternatively, we performed the siRNA experiments using the WT8 cells transfected to express either MT1-MMP or HIF-2α in order to mimic the ability of the pRc-9 cells to degrade collagen (see Figures 1B, 2A, 2C). Specifically, we co-transfected the WT8 cells with MTpc3SE and one of three specific MT1-MMP siRNAs or a non-specific control siRNA and measured MT1-MMP mediated collagen invasion. GFP expression was used to measure transfection efficiency and to track tumor cell invasion using FluoroBlok™ membranes as described below. A dose response analysis showed that the concentration of siRNA oligos that reduced MT1-MMP mRNA by >50% was 25 nM (data not shown). The reduction in MT1-MMP protein levels by the specific siRNAs was confirmed with an ELISA assay and was >60% as compared to the control siRNA (*P *< 0.005; Figure 3A).

In addition to its function as a matrix-degrading enzyme, MT1-MMP also acts as a cell surface receptor for pro-MMP-2 and promotes MMP-2 activation [46-48]. Specifically, MT1-MMP, TIMP-2 and pro-MMP-2 exist in a trimolecular complex at the cell membrane [48]. When MT1-MMP levels are in excess of TIMP-2 and pro-MMP-2, a free MT1-MMP molecule cleaves the propeptide sequence of pro-MMP-2 (72 kDa), thereby generating the intermediate (69 kDa) form [49]. Fully active (67 kDa) MMP-2 is achieved by either intermolecular autolytic cleavage or by the activity of other enzymes [46,47,50]. WT8 cells express low levels of MMP-2 as well as TIMP-2 [23,34]. To confirm that the reduction in MT1-MMP protein by siRNA was reflected in an inhibition of MT1-MMP enzyme activity, we measured the presence of pro, intermediate, and active forms of MMP-2 in the conditioned media collected from the transfectants using gelatin zymography. Expression of MTpc3SE in the WT8 cells resulted in the presence of the intermediate and active forms of MMP-2 (Figure 3B). Inhibition of MT1-MMP activity by each MT1-MMP siRNA was demonstrated by the absence of the MMP-2 intermediate and active species in these samples as compared to the media from the siRNA control transfectants (Figure 3B). The only other MMPs constitutively expressed by these cells in addition to MT1-MMP are MMP-2 and MMP-9 [23]. Therefore, it is important to note that the MT1-MMP siRNAs have no effect on the expression levels of MMP-2 or MMP-9 as seen by the unchanged levels of the bands representing these proteins in Figure 3B, confirming the specificity of these siRNAs against MT1-MMP.

Next, the ability of the siRNA transfectants to invade through a type I collagen matrix was measured using a similar *in vitro *invasion assay system described in the previous Figures. For these invasion assays, we used type I collagen-coated FluoroBlok™ membrane transwell inserts. FluoroBlok™ membranes block the transmission of light; therefore, non-invasive cells are not detectable, whereas invasive, pCMV-eGFP transfected cells can be counted by fluorescence imaging. By only visualizing GFP-expressing cells that have invaded, we could specifically quantitate the number of transfected cells that had invaded collagen while discounting the non-transfected cells. As before (see Figure 2D), WT8 cells expressing exogenous MT1-MMP displayed increased collagen invasion (*P *< 0.005; Figure 3C). Co-transfection of the control siRNA oligo had no significant effect on this increased invasion by MT1-MMP, whereas each of the three specific MT1-MMP siRNA oligos inhibited collagen invasion of the MT1-MMP transfectants by greater than 85% (Figure 3C). Taken together, these data suggest that MT1-MMP is important for tumor cell invasion by RCC cells.

**Figure 4:**
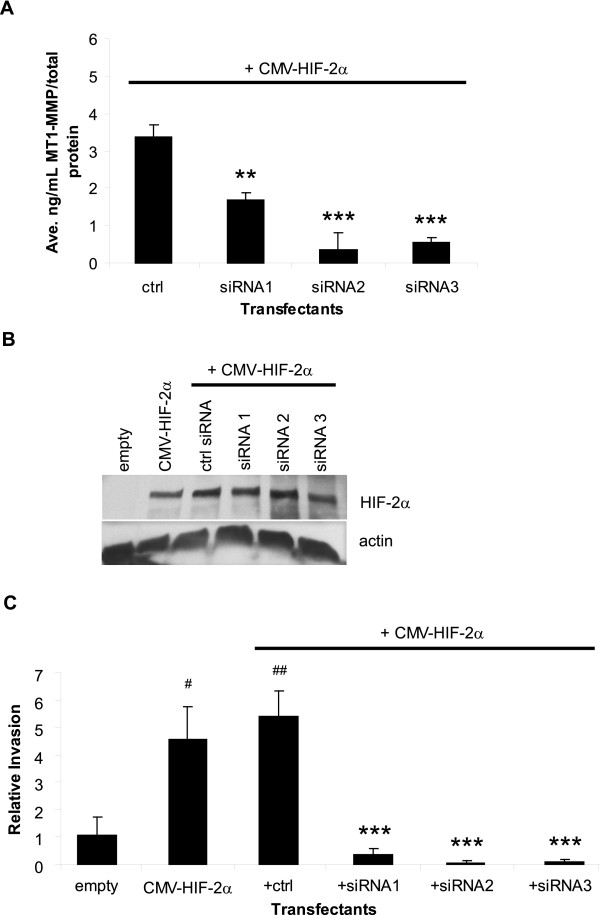
**Specific inhibition of MT1-MMP blocks HIF-2α mediated RCC tumor cell invasion**. **A**. MT1-MMP protein expression as quantitated by an MT1-MMP ELISA activity assay. WT8 cells were transfected for 48 hours with pCMV-HIF-2α and a control or 3 specific MT1-MMP siRNA oligos. Each transfectant was additionally co-transfected with pCMV-eGFP. Transfection efficiency was consistent among the transfectants and was approximately 50%. Values represent the average [ng/mL] MT1-MMP of three transfections normalized to [μg/mL] total protein and are representative of three experiments (mean +/-S.D.); *P *< 0.005(**), *P *< 0.0005 (***) compared to the control siRNA. **(B) and (C) **WT8 cells were transfected for 48 hours with a control empty vector, pCMV-HIF-2α alone, or pCMV-HIF-2α and control or 3 specific MT1-MMP siRNA oligos. Each transfectant was additionally co-transfected with pCMV-eGFP. Transfection efficiency was approximately 50%. **B**. Western blot analysis of HIF-2α protein from whole cell lysates of the transfectants. Actin was used as a loading control. **C**. *In vitro *invasion assay of the transfectants using type I collagen coated FluoroBlok™ membrane inserts as described in Fig 3. Invaded cells were viewed by GFP fluorescence and counted from the entire membranes at 40×. Values represent the average number of invaded cells from three separate transfections set as relative invasion of the empty vector control and are representative of two experiments (mean+/-S.D.); *P *< 0.05 (#),*P *< 0.005 (##) compared to empty vector control; *P *< 0.0005 (***) compared to control siRNA. Statistical analyses were performed using the student's *t*-test.

We previously identified MT1-MMP as a target of HIF-2α [23], and our current data show that expression of HIF-2α in a wild-type VHL background induces the ability of the RCC cells to degrade type I collagen (see Figure 2A). Together these results suggest that the increase in collagen destruction mediated by HIF-2α overexpression in the WT8 cells is due to the induction of MT1-MMP expression. To test this hypothesis, we transfected the WT8 cells with pCMV-HIF-2α and either the control siRNA oligo or one of the three MT1-MMP siRNAs. As shown in Figure 4A, MT1-MMP protein levels were reduced by the target siRNAs in the HIF-2α transfectants by at least 50%. To ensure that the inhibition of MT1-MMP expression by the siRNAs was not due to non-specific effects of the siRNAs on HIF-2α protein levels, we measured HIF-2α protein in the transfectants. The WT8 cells do not endogenously express detectable levels of HIF-2α protein due to the presence of VHL [17] as shown in the empty vector control (Figure 4B); however, HIF-2α protein is detectable in these cells upon transfection of CMV-HIF-2α, and the MT1-MMP siRNAs had no effect on HIF-2α protein. Next, we employed the FluoroBlok™ invasion assay system described for Figure 3C to measure the ability of these transfectants to invade type I collagen. Inhibition of MT1-MMP blocked HIF-2α mediated invasion of these RCC cells by >90% (Figure 4C). To our knowledge, the only other HIF-2α target gene identified in playing a role in RCC tumor cell migration and invasion is the chemokine receptor, CXCR4 [51]. No effect of the MT1-MMP siRNAs on the expression of CXCR4 was detected in the HIF-2α transfectants (data not shown); thus, HIF-2α mediated cell invasion is blocked by specific inhibition of MT1-MMP. Together, these data imply that MT1-MMP is the HIF-2α target gene responsible for the increased tumor cell invasion observed in VHL mutant RCC cells.

## Discussion

Renal cell carcinoma cells that have lost VHL tumor suppressor function lose the ability to negatively regulate HIF-2α protein levels, thereby allowing HIF-2α protein to accumulate and dimerize with HIF-β to form a functional transcription factor [17]. Consequently, downstream targets of HIF, such as MT1-MMP, are constitutively transcribed, and activation of these targets causes RCC tumor formation in xenograph models [20-22]. Defining the HIF-2 targets responsible for VHL null RCC tumor progression is important to understand the mechanisms of renal tumor development. It is not yet known whether elevated expression of MT1-MMP contributes to RCC tumorigenesis or progression to metastatic disease.

When compared to several of the secreted MMPs, MT1-MMP and MT2-MMP are the only MMPs able to confer collagen invasive capabilities to non-invasive cells, thereby suggesting that pericellular proteolysis is critical for tumor cell migration and invasion [52]. Furthermore, specific inhibition of MT1-MMP in tumor cells overexpressing MT1-MMP is sufficient to suppress tumor cell migration, invasion, proliferation, and metastasis [53-55]. Recently, the importance of MT1-MMP in cancer progression has been demonstrated by the finding that overexpression of MT1-MMP in non-malignant cells was sufficient to drive tumorigenicity [56]. In keeping with these findings, we previously described a mechanism of MT1-MMP transcriptional upregulation in VHL mutant RCC cells and hypothesized that MT1-MMP may play a role in the invasion of renal cell carcinoma [23].

Here, we provide evidence supporting the importance of MT1-MMP in the invasive properties of RCC cells. First, our data show that RCC cells null for VHL (pRc-9) have a greater propensity for collagen degradation and invasion than RCC cells wild-type for VHL (WT8), suggesting that the pRc-9 cells have increased invasive potential (Figure 1). Further, overexpression of HIF-2α or MT1-MMP was sufficient to confer increased degradative and invasive abilities to the WT8 cells in type I collagen (Figure 2). Finally, inhibition of collagen invasion by WT8 cells transfected to express either MT1-MMP or HIF-2α required specific inhibition of MT1-MMP expression (Figures 3, 4). Importantly, collagen invasion of the HIF-2α transfectants was almost completely abrogated (>90%) by the MT1-MMP siRNAs, suggesting that MT1-MMP is the HIF-2α target primarily responsible for collagen invasion by these cells.

The process of matrix invasion by a tumor cell requires both degradation of ECM components as well as cell migration [39]. In the pRc-9 cells, migration may result from the lack of VHL, which regulates migration through various mechanisms, including the regulation of actin filaments [42], integrin fibrillar adhesions [57], and endocytosis of FGFR1 [45]. By performing siRNA experiments in a wild-type VHL background, we determined that invasion of the RCC cells was dependent on MT1-MMP rather than on the dysregulation of other VHL targets, suggesting that MT1-MMP may be responsible for RCC tumor invasion in the stromal compartment.

Our siRNA studies showed that inhibition of MT1-MMP expression prevented the activation of pro-MMP-2 as a measure of functional inhibition of MT1-MMP (Figure 3B) [46-48]. Thus, it is plausible that MT1-MMP may also contribute to RCC invasion of basement membrane through its regulation of pro-MMP-2 activation since MMP-2 degrades type IV collagen, the main component of basement membrane [33]. Interestingly, expression MMP-2 has been correlated with advanced stages of RCC, and MMP-2 activity, along with histological grade, stage, and T classification, has been identified as a significant predictor of RCC clinical outcome [58-60].

Since the expression of the MT1-MMP siRNAs inhibited the activation of pro-MMP-2 by MT1-MMP (Figure 3B), our data does not exclude the possibility that MMP-2 activity may be required for type I collagen invasion by these RCC cells. However, this situation seems unlikely. Although MMP-2 has been shown to cleave fibrillar type I collagen, its ability to do so is less effective than known collagenases and requires a cell-free, TIMP-free system [71], which was not the condition under which we performed our experiments. In particular, the WT8 cells used in the siRNA studies (Figures 3, 4) express TIMP-1 and TIMP-2, as previously described [34]; thus, MMP-2 may not cleave fibrillar type I collagen under our experimental conditions. Further, the intermediate form of MMP-2, a species generated only when MT1-MMP and TIMP-2 are present at specific ratios, is predominant in these cells (Figure 3C) [49]. Thus, it is unlikely that in our system, MMP-2 is playing a substantial role in the type I collagen invasion by these cells. Nonetheless, since MMP-2 activity is dependent on MT1-MMP [46-48], we conclude from our data that MT1-MMP is required for type I collagen invasion by these RCC cells. Thus, we posit that MT1-MMP may initiate local invasion by RCC tumors by promoting both cell migration and invasion through cleavage of adhesion molecules, such as CD44 and integrins, pro-MMP-2, and by pericellular proteolysis of type I collagen fibrils in the stromal environment [30,61]. Supporting this hypothesis, MT1-MMP mRNA expression is increased in invasive RCC tumors when compared to tumors that remain localized [31].

Our experiments using HIF-2α expression in the WT8 cells demonstrate that HIF-2α increased type I collagen invasion despite the presence of wild-type VHL, and this increased invasion was dependent on MT1-MMP (Figure 4C). Kurban *et al*. recently showed that HIF-2α expression in a VHL wild-type background does not promote invasion of Matrigel [38], a reconstituted basement membrane mainly comprised of type IV collagen and laminin [62]. The authors concluded from their studies that invasion by VHL mutant cells is mediated via a HIF-independent mechanism, namely, by the loss of ECM assembly. In their Matrigel invasion assays, Kurban *et al*. used the RCC cell line, WTPA, which stably expresses a HIF-2α variant that is not degraded by VHL in a VHL wild-type background [20]. In their study, WTPA cells did not invade Matrigel as effectively as VHL mutant cells. Importantly, the authors showed that the more invasive cell lines expressed MMP-2, whereas WTPA cells did not. In our previous studies, we showed that pRc-9 and WTPA cells have stabilized HIF-2α protein and high levels of MT1-MMP when compared to WT8 cells [23]. Like Kurban *et al*., we found that MMP-2 expression in WTPA cells was similar to WT8 cells and much lower than pRc-9 cells (data not shown), suggesting that the mechanism of MMP-2 overexpression in pRc-9 cells may not be HIF-2α dependent. Furthermore, invasion of Matrigel likely requires MMP-2 gelatinolytic activity through the degradation of type IV collagen, whereas type I collagen invasion, as in our assays, may not require MMP-2 activity. Taken together, we conclude that while RCC cell invasion of Matrigel may be HIF-independent, HIF-2α mediates type I collagen of RCC cells through the regulation of MT1-MMP.

## Conclusion

Metastatic renal cell carcinoma remains difficult to treat. Although IL-2 and IFN-γ are the standard of care for these patients, the response rate is very low [4,63]. The VHL-HIF pathway is a well-defined link to clear cell renal cell carcinogenesis, and thus targeting this pathway may benefit VHL RCC patients [64,65]. Many next-generation agents currently in clinical trials for RCC are therapies targeting proteins regulated by the VHL-HIF pathway, such as VEGF and TGF-α [63,64,66]. We provide evidence supporting an important role for MT1-MMP in VHL RCC tumor cell invasion of type I collagen. We conclude that the loss of VHL tumor suppressor function is mechanistically linked to invasive behavior through the regulation of MT1-MMP, thereby implicating MT1-MMP as a potential therapeutic target for the treatment of invasive RCC.

## Methods

### Cell lines and cell culture

Cell lines were maintained in Dulbecco's Modified Eagle's Medium (Mediatech, Inc., Herndon, VA) supplemented with 10% fetal bovine serum (FBS) (Hyclone, Logan, UT), penicillin [100 U/mL], streptomycin [100 μg/mL], and L-glutamine and cultured at 37°C, 5%CO_2_. For serum-free conditions, DMEM supplemented with lactalbumin hydrosylate (2%), penicillin [100 U/mL], streptomycin [100 μg/mL], and L-glutamine was used. The WT8 and pRc-9 cell lines (kindly provided by William Kaelin, Dana-Farber Cancer Institute, Boston, MA) represent stable subclones of the 786-0 renal cell carcinoma cell line transfected with pRc/CMV-HA-VHL or pRc/CMV-empty, respectively [16]. The 786-0 cell line (American Type Culture Collection (Manassas, VA) has a single VHL allele harboring a frameshift mutation at codon 104 resulting in a truncated, non-functional protein [16,67]. The WT8 and pRc-9 cells were cultured under continuous selection with the addition of [1 mg/mL] G418 sulfate. Cells were washed with either Hank's Balanced Salt Solution (HBSS) (Mediatech, Inc.) or 1× PBS (13.7 mM NaCl, 0.27 mM KCl, 1.2 mM phosphate buffer, pH 7.4) (National Diagnostics, Atlanta, GA) as indicated in the different experimental procedures.

### Reagents and plasmids

The pCMV-HIF-2α expression construct containing full-length cDNA of HIF-2α was a generous gift of Richard Bruick (University of Texas, Southwestern Medical Center, Dallas, TX) [68]. The MT1-MMP expression construct, MTpc3SE, was a kind gift of Jouko Lohi (University of Helsinki, Helsinki, Finland), and contains full-length MT1-MMP cDNA downstream of a CMV promoter [69]. The pCMV-eGFP expression construct is commercially available from BD Biosciences (San Jose, CA).

### Transient transfections

WT8 cells were plated in 6-well dishes at a density of 1.5 × 10^5 ^cells/well in DMEM+10% FBS in the absence of antibiotics or selection. At this density, the cells were ~90% confluent the following day and ready for transfection. Cells were then transiently transfected with 1 μg of DNA using Lipofectamine 2000™ Transfection Reagent (Invitrogen, Carlsbad, CA) following manufacturer's instructions for at least 12 hours before being washed three times with HBSS and switched to serum-free conditions for additional time depending on the experiment. Transfection with an empty vector (pRc/CMV) was used as a control for mock transfected cells [23]. All transfections were performed in triplicate. Separate plates of cells were transfected with pCMV-eGFP to control for transfection efficiency under the given conditions. Transfection efficiency was determined as a percentage of GFP-expressing cells counted from 3 fields of 3 separate transfections, and the average transfection efficiency was approximately 50% in all experiments. Cells were harvested for either 1) functional assays as described under 'Collagen invasion assays' or 'Collagen degradation assay', 2) total RNA using the RNeasy kit as described under 'Real-time RT-PCR', or 3) protein expression analyses as described under 'ELISA' or 'Immunoblotting'.

### siRNA transfections

Three siRNA duplex oligoribonucleotides targeting the MT1-MMP coding region (NM_604995) were designed using the Block-iT™ RNAi Designer program from the Invitrogen website. The Stealth™ RNAi Negative Control Duplex (cat# 12935-300; Invitrogen, Carlsbad, CA) is a proprietary non-targeting sequence with medium G/C content, which is similar to the G/C content of the target Stealth™ siRNAs. The target siRNA sequences are as follows: *siRNA(1)*: 5'-AAUUUGCCAUCCUUCCUCUCGUAGG-3'; *siRNA(2)*: 5'-AAGAGAGCAGCAUCAAUCUUGUCGG-3'; *siRNA(3)*: 5'-AAUGAUGAUCACCUCCGUCUCCUCC-3'. WT8 cells were plated in 6-well dishes at a density of 1.5 × 10^5 ^cells/well as described under 'Transient transfections'. The following day, cells were transfected with 0.5 μg of pCMV-eGFP, 1 μg MTpc3SE or pCMV-HIF-2α, and 25 nM of either the control siRNA or one of the MT1-MMP target siRNAs using Lipofectamine 2000™ Transfection Reagent (Invitrogen) following manufacturer's instructions for co-transfection of plasmid DNA and siRNA oligos. Cells were transfected in the presence of serum for 24 hours before being washed three times with HBSS and switched to serum-free conditions for an additional 24 hours. Transfection efficiency was determined by GFP expression as described above before the cells were harvested for either protein expression or the collagen invasion assay as described under 'Fluoroblok™ invasion assay'. The average transfection efficiency was approximately 50% in all experiments. An empty vector control (pRc/CMV) was used to balance the amount of plasmid DNA in co-transfections [23]. All transfections were performed in triplicate.

### Real-time RT-PCR

Total cellular RNA was purified using the RNeasy kit with on-column DNase I treatment (Qiagen, Valencia, CA). Reverse transcription and real-time PCR reactions were performed using the Taqman Reverse Transcription Reagent Kit and Syber Green Master Mix, respectively, following the manufacturer's protocol and as described previously (Applied Biosystems, Foster City, CA) [23]. For each experiment, triplicate samples were obtained, and the cDNA for each was assayed in duplicate using a MJ Research DNA Engine Opticon thermal cycler. Data are presented as the average pg of MT1-MMP mRNA per ng of GFP mRNA and are representative of three or more experiments. Standard curves were included in each assay. Standards were prepared from serial log dilutions of plasmids carrying the appropriate cDNA as follows: MTpc3SE plasmid, 10 pg-0.001 pg; pCMV-eGFP plasmid, 1 ng-0.001 ng. Primer sequences are as previously published for MT1-MMP [23] and for eGFP [41].

### ELISA

The Matrix Metalloproteinase-14 (MMP-14) Biotrak Activity Assay System (GE Healthcare BioSciences Corp., Piscataway, NJ), a quantitative measure of MT1-MMP activity as a direct measure of MT1-MMP protein, was used to analyze MT1-MMP protein expression. The assay was conducted according to the manufacturer's protocol. A standard curve ranging from [0.125 ng/mL] to [8 ng/mL] was used to quantitate protein expression. Because the assay is colorimetric and does not require quenching, absorbance readings were taken at 6 hr, 9 hr, and 12 hrs after incubation with the substrate. The data presented represent the concentration of MT1-MMP in the samples at the timepoint at which the sample values fit the standard curve most appropriately. Total protein from the extracts was quantitated using the Bradford Assay (Bio-Rad, Hercules, CA) and used to normalize the MT1-MMP protein concentration in each sample.

### Immunoblotting and antibodies

Whole cell lysates were harvested from transfection experiments by washing the cells twice with cold 1× PBS, adding 100 μL of SDS reducing buffer (60 mM Tris-HCl, pH 6.8, 2% SDS, 14.4 mM β-mercaptoethanol, 25% glycerol, 0.1% bromphenol blue), and boiling for 5 minutes. Proteins were resolved by SDS-PAGE and electrotransferred to Immobilon-P PVDF membranes (Millipore Corp., Bedford, MA). Membranes were blocked with 5% milk in Tris-buffered saline 0.1% Tween-20 at room temperature for 1–2 hours. Primary antibodies, HIF-2α polyclonal antibody, 1:1000 (Novus Biologicals, Littleton, CO) and actin monoclonal antibody, 1:5000 (Oncogene, Cambridge, MA), were diluted in blocking buffer and incubated with the membranes at room temperature for 2 hours. Appropriate secondary antibodies were diluted in blocking buffer and incubated with the membrane at room temperature for 1 hour. Proteins were visualized by Western Lightning Chemiluminescence Reagent (Perkin Elmer, Boston, MA).

### Gelatin Zymography

Transfectants were cultured in serum-free media for 24 hours, and conditioned media was concentrated approximately 30 fold using BioMax 30 K NMWL membrane ultrafree filters (Millipore Corp., Bedford, MA) as per manufacturer's instructions. An equal volume of non-reducing buffer (60 mM Tris-HCl, pH 6.8, 25% glycerol, 0.1% bromphenol blue) was added to the concentrated media before being applied to a 10% SDS-PAGE gel impregnated with [2.8 mg/mL] gelatin. After electrophoresis, the gel was washed two times for 30 min in 50 mM Tris-HCl (pH 7.5), 5 mM CaCl_2_, 5 μM ZnCl_2_, plus 2.5% Triton X-100 followed by an overnight incubation at 37°C in the same buffer without Triton X-100. Gels were stained with Coomassie Brilliant Blue R-250 and then destained in 20% methanol, 10% glacial acetic acid. Activity was detected as transparent bands. Recombinant human pro-MMP-2 (R&D Systems, Minneapolis, MN) was used as a control for gelatinolytic activity at [1 ng/μL]. Active MMP-2 was obtained by incubation with 20 μM 4-Aminophenylmercuric acetate (APMA, Sigma, St. Louis, MO) at 37°C for 45 min.

### Collagen invasion assays

#### Transwell^® ^invasion assay

Cells were serum-starved overnight before being harvested for invasion assays. Transwell^® ^inserts (Costar, 24 mm, Corning, NY) with 8.0 μm pore polycarbonate membranes were coated with 50 μL per cm^2 ^growth area with type I collagen diluted to [1 mg/mL] in serum-free DMEM. Collagen was prepared from purified bovine type I collagen (Cohesion Technologies, Palo Alto, CA) following manufacturer's instructions. Briefly, collagen was neutralized to pH 7.4 with the addition of 10× PBS and 0.1 N NaOH. The collagen was allowed to gel on top of the membrane at 37°C for 1 hour. Next, either DMEM+10% FBS or serum-free DMEM was added to the lower chamber as a chemoattractant. Cells were plated on top of the collagen layer at a density of 3.0 × 10^5 ^cells per insert in serum-free media. Cells were allowed to invade for 4–6 hours. At time of harvest, media in the upper chamber was removed, and the upper surface of the membranes was scraped with a cotton swab to remove the collagen gel and remaining cells. The membranes were rinsed with 1× PBS and then removed from the inserts using a scalpel. The membranes were then stained with methylene blue and destained with water. Stained cells were viewed using an Olympus 1 × 50 inverted phase contrast microscope at 100× and counted from 3 fields. Pictures were taken with an Olympus Q Color 3 camera.

#### FluoroBlok™ invasion assay

siRNA transfected cells were serum-starved for 24 hours before being harvested for the invasion assay. HTS FluoroBlok™ inserts (BD Falcon Labware, 6.5 mm, Franklin Lakes, NJ) contain fluorescence blocking PET track-etched membranes with 8.0 μm pores. This invasion system allowed for real-time viewing of cell invasion without the need to end the experiment and process the membranes. The FluoroBlok™ membrane prevents the transmission of light to cells on top of the membrane; thus, only invaded, GFP-expressing cells invaded through the collagen-coated membrane can be viewed. FluoroBlok™ membranes were coated with 90 μL per cm^2 ^growth area with type I collagen diluted to [0.8 mg/mL] in serum-free DMEM. Collagen was prepared from purified bovine type I collagen (Organogenesis, Inc., Canton, MA) and neutralized to pH 7.4 with a buffer containing 9.8% 10× EMEM, [200 nM] L-Glutamine, 2% lactalbumin hydrosylate, 7.5% sodium bicarbonate. The collagen was allowed to gel on top of the membrane at 37°C for 30 min. Next, DMEM+10% FBS was added to the lower chamber as a chemoattractant. Cells were plated on top of the collagen layer at a density of 2.1 × 10^4 ^cells per insert in serum-free DMEM. Invaded cells were viewed by GFP fluorescence using excitation from a 100 W Mercury lamp on an Olympus 1 × 50 inverted phase contrast microscope. Cells were counted from entire membranes at 40×.

### Collagen degradation assay

Cells were serum-starved for at least 8 hours before being harvested for the assay. Cells were embedded as a mixture of fibrillar collagen and media in a 12-well assay format. Collagen was prepared from purified bovine type I collagen (Cohesion Technologies, Palo Alto, CA) following manufacturer's instructions. Briefly, collagen was neutralized to pH 7.4 with the addition of 10× PBS and 0.1 N NaOH. Next, 3.2 × 10^5 ^cells were mixed with 132 μL of neutralized collagen, and serum-free media was added to bring the total volume to 635 μL per well. The final concentration of collagen in the mixture was [0.5 mg/mL]. The collagen was allowed to gel for 1 hour at 37°C after which 635 μL of serum-free media was added to the top of the gel. Assays were harvested at 48 hours, and the overlying media was removed and weighed. The specific gravity of serum-free media was determined to be 1 mg/mL. Therefore, collagen degradation is reported as the volume of liberated media calculated by the difference of the weight of total media removed and weight of the original volume added (635 μg or 635 μL). This assay provides an accurate measure of collagen degradation *in vitro *[40,41].

### Statistical analysis

Statistical significance was calculated using the student's *t*-test available online [70] and are represented as +/- standard deviation (S.D.) of the mean. Significance was assigned to *P *values < 0.05.

## Competing interests

The author(s) declare that they have no competing interests.

## Authors' contributions

B.L.P. contributed to the design of the study, acquisition of the data presented, data analysis, and the writing of the manuscript. C.E.B. contributed to the design of the study, data analysis, and critical reading of the manuscript.
